# Acute liver injury in the context of immune checkpoint inhibitor-related colitis treated with infliximab

**DOI:** 10.1186/s40425-019-0532-1

**Published:** 2019-02-18

**Authors:** Hao Chi Zhang, Wenyi Luo, Yinghong Wang

**Affiliations:** 1 Division of Gastroenterology, Hepatology and Nutrition, The University of Texas Health Science Center at Houston, Houston, TX 77030 USA; 2 Department of Pathology/Laboratory Medicine, The University of Texas MD Anderson Cancer Center, Houston, TX 77030 USA; 3 Department of Gastroenterology, Hepatology and Nutrition, The University of Texas MD Anderson Cancer Center, 1515 Holcombe Blvd., Unit 1466, Houston, TX 77030 USA

**Keywords:** prostate cancer, immune checkpoint inhibitors, infliximab, liver injury

## Abstract

**Background:**

Immune checkpoint inhibitors (ICPIs), used to treat different advanced malignancies, are associated with a wide range of immune-related adverse reactions (irAEs) that deserve close monitoring of patients. Gastrointestinal reactions and hepatotoxicity may occur, which warrant careful evaluation to confirm the etiology and attribution to ICPIs as these events could affect future management.

**Case presentation:**

We describe a case of a patient with prostate adenocarcinoma, treated with dual ICPIs comprised of ipilimumab and nivolumab, who developed elevated liver enzymes in the context of infliximab therapy prescribed to treat gastrointestinal irAE from his ICPIs. The patient’s grade 3 colitis became steroid-refractory, requiring a one-time infusion of infliximab, a biologic agent used commonly in inflammatory bowel disease, as a rescue therapy, to which he responded. The patient subsequently developed liver injury. This presented a diagnostic dilemma involving differential diagnoses of hepatotoxicity due to ICPI or infliximab exposure. A careful review of the clinical history, evaluation of the chronology of events, and exclusion of other causes of acute hepatitis were employed to make the final diagnosis of this event as infliximab-associated hepatotoxicity.

**Conclusion:**

ICPIs such as CTLA-4 and PD-1 inhibitors have the potential to cause both gastrointestinal reactions and hepatotoxicity. An additional confounding factor in our patient’s case was the exposure to infliximab used to manage an established irAE that developed after the last exposure to ICPIs. The clinical history and data supported infliximab-associated hepatotoxicity, rather than an irAE. With the increasing application of ICPIs for different cancers, in conjunction with potential risks for irAE, the liver profile should be closely monitored during treatment with ICPI as well as with anti-TNF-α agents in this patient population.

## Background

Immune checkpoint inhibitors (ICPIs) such as ipilimumab, a CTLA-4 inhibitor, and nivolumab, a PD-1 inhibitor, are more widely used to treat various types of cancers, but they can also be associated with immune-related adverse reactions (irAEs) including enterocolitis, hepatitis, or skin rash. Careful attribution of irAEs is important as it may impact the patient’s subsequent cancer therapy. The development of liver injury secondary to ICPI administration is infrequent but well-known and reported. The use of infliximab as an agent for steroid-refractory colitis secondary to ICPI has been reported and potentially effective. However, infliximab is also associated with hepatotoxicity. We describe a case with a complicated history that prompted careful analysis of the differential diagnoses for acute liver injury presented with new elevation in liver enzymes.

### History of present illness

A 79-year-old man with a history of metastatic prostate adenocarcinoma, treated with ICPIs ipilimumab and nivolumab, and enterocolitis as irAE treated with infliximab, was evaluated for new elevation in liver enzymes and bilirubin.

The patient had a past medical history including essential hypertension and hypertriglyceridemia. He recently recovered from influenza A H3 virus infection. He was also treated for a skin rash which was deemed to be an adverse event from ICPI. Otherwise, there was no history of chronic liver disease or diabetes. Prior imaging of the abdomen before exposure to ipilimumab and nivolumab did not reveal evidence of hepatosteatosis. He consumed alcohol weekly, including three glasses of wine and one can of beer per week. Family history included colon cancer of the patient’s father and brother.

Previous management of his prostate cancer involved neoadjuvant chemotherapy with temsirolimus during the initial year of diagnosis 13 years prior, followed by radical prostatectomy and pelvic lymph node dissection. He underwent intermittent androgen deprivation therapy with leuprolide within a year after diagnosis, during which time he was diagnosed with pelvic lymph node metastasis in 5 years thereafter. Given pelvic and right femur metastases, he underwent palliative radiotherapy to the right femur. He was also treated with a period of abiraterone acetate with prednisone before this presentation during the same year. As he had demonstrated progression of disease, ICPIs were initiated.

**Fig. 1:**
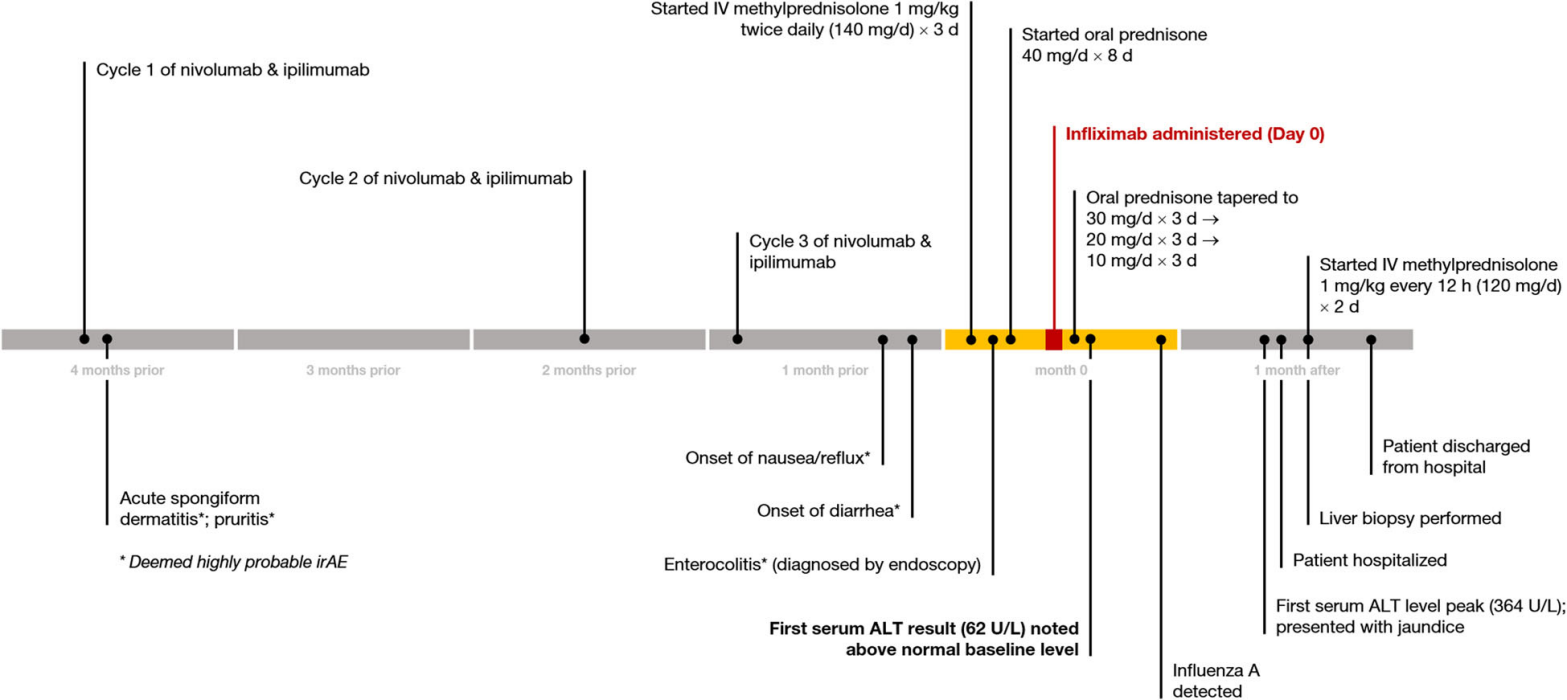
Chronology of history of present illness, delineating key points related to medication administration, clinical events, and detection of liver enzyme abnormalities

The patient underwent institutional Phase 2 protocol treatment (2016–0848) with combination regimen of ipilimumab 1 mg/kg and nivolumab 3 mg/kg (for body weight of 73 kg), starting just over 4 months prior to this presentation (Fig. 1). The second cycle was administered 9 weeks after the first cycle, and the third cycle was administered 3 weeks thereafter (Fig. 1). Shortly after the third cycle, the patient began to suffer from loose bowel movements, progressing to acute frequent non-bloody diarrhea characterized as four episodes during the day and up to twelve nocturnal episodes. This diarrhea was also associated with anterior abdominal discomfort and acute acid reflux symptoms. Due to concern for grade 3 irAE of diarrhea/colitis, the patient’s ICPIs were discontinued at that point.

### Evaluation of gastrointestinal irAE

A contrast-enhanced computed tomography (CT) of the abdomen and pelvis at the time showed evidence of mild wall thickening of the distal ileal loops, and stable finding of prior small low-attenuation liver lesions thought to be cysts, without biliary ductal dilatation. *Clostridium difficile* testing and gastrointestinal enteric pathogen testing were negative for infectious causes of diarrhea. Fecal calprotectin was elevated at 484 μg/g (reference range: ≤50 μg/g).

**Fig. 2:**
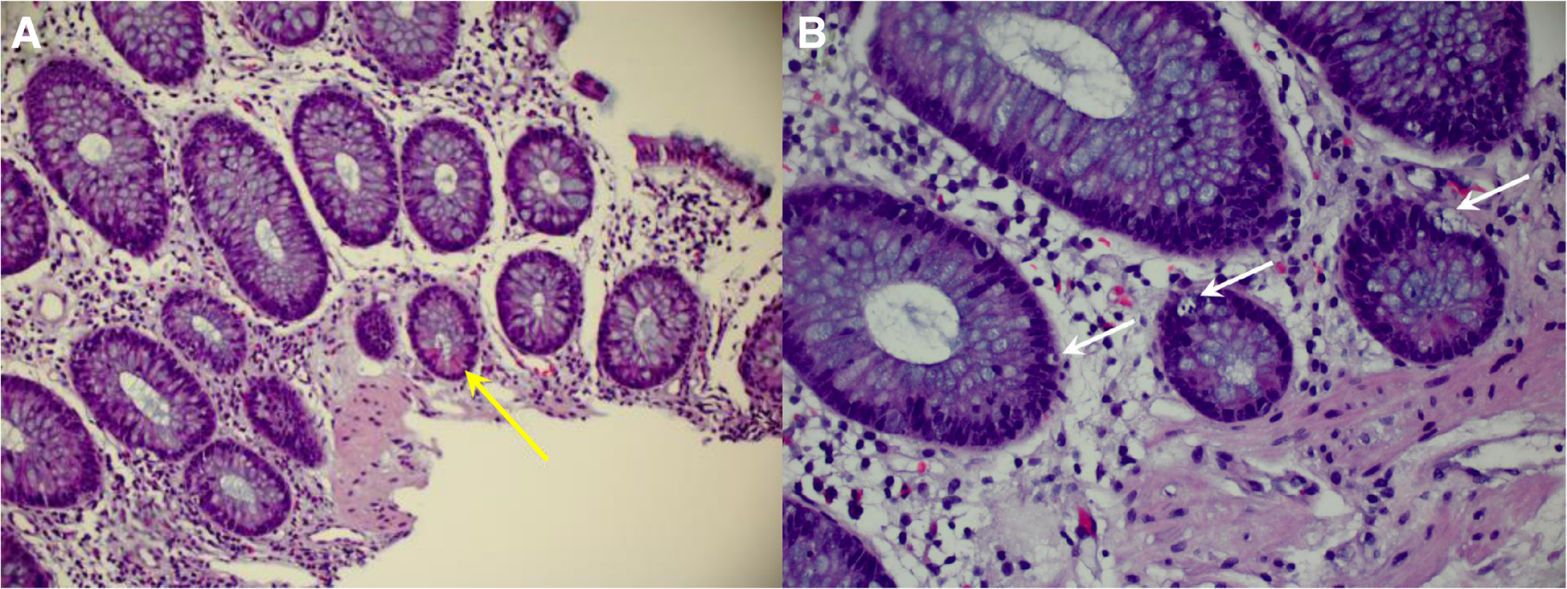
Biopsies from colonic mucosa. Panel **a**: (Hematoxylin and eosin stain, 20×) Paneth cell metaplasia (yellow arrow). Panel **b** (Hematoxylin and eosin stain, 40×) Increased apoptosis (white arrows)

An upper endoscopy revealed small erosions in the distal gastric body and pre-pyloric region, normal-appearing duodenum, and no gastroesophageal varices; biopsies revealed duodenitis and chronic inflammation in the stomach. Ileo-colonoscopy revealed mild erythema of the terminal ileum as well as mild to moderate erythema in the entire colonic mucosa with normal-appearing rectum; biopsies revealed diffuse chronic mucosa injury and increased apoptosis (Fig. 2), most compatible with ICPI-associated enterocolitis. No further ipilimumab or nivolumab was given to this patient.

### Hepatotoxicity event

For management of gastrointestinal irAE, high-dose intravenous methylprednisolone (1 mg/kg twice daily, for body weight of 67 kg) was started (Fig. 1), leading to mild improvement in diarrhea after 3 days. The steroid regimen was then transitioned to prednisone 40 mg/d for the next 8 days (Fig. 1).

**Fig. 3:**
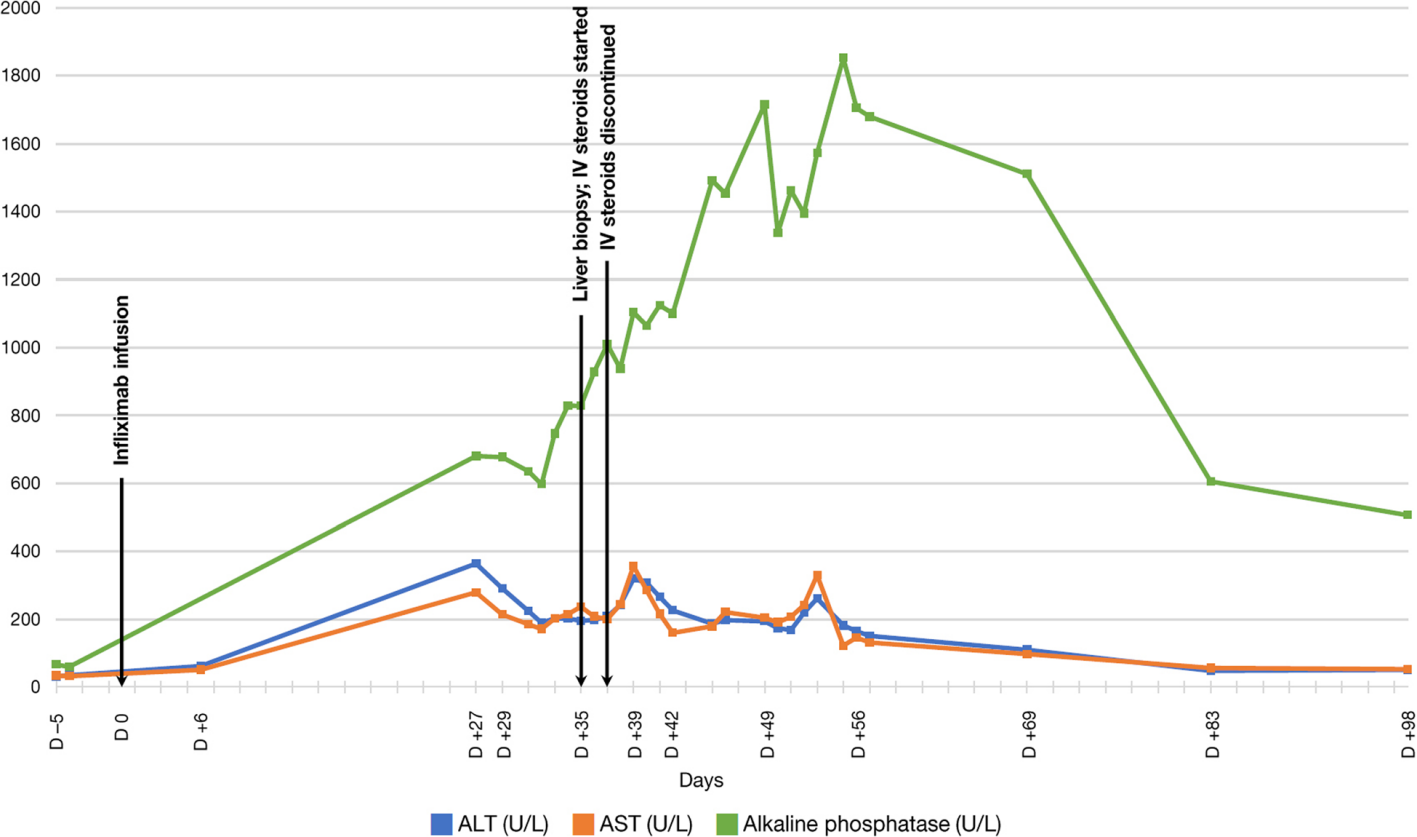
Trends in results of liver biochemical testing (serum ALT, AST, alkaline phosphatase levels), displayed in days relative to infliximab infusion (lines connect available data points)

**Fig. 4:**
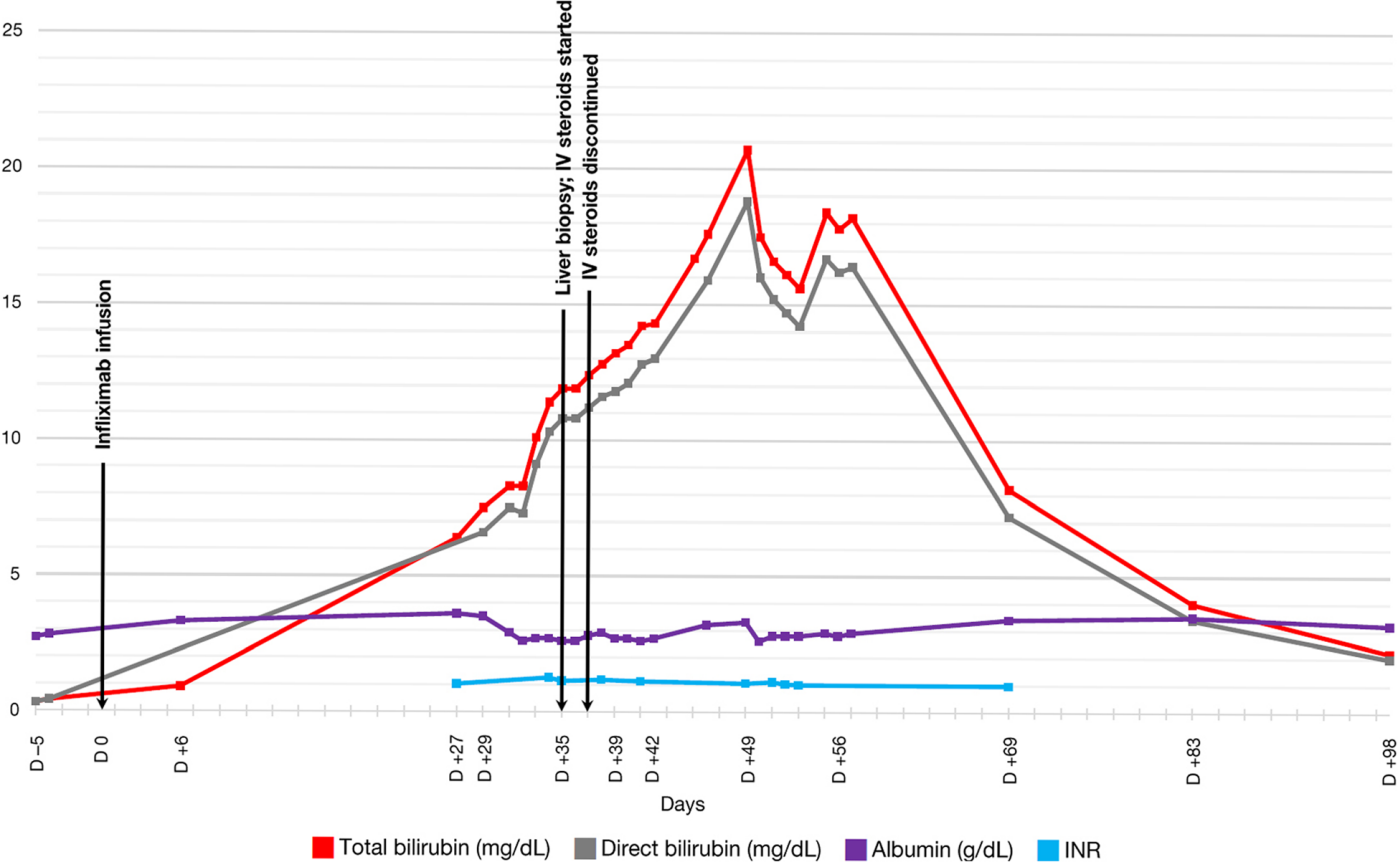
Trends in results of liver biochemical testing (total bilirubin, direct bilirubin, albumin, and INR), displayed in days relative to infliximab infusion (lines connect available data points)

The patient’s liver biochemical testing after brief hospitalization prior to discharge included serum ALT 35 U/L (reference range: 7–56 U/L), AST 32 U/L (reference range: 15–46 U/L), alkaline phosphatase (ALP) 60 U/L (reference range: 38–126 U/L), total bilirubin 0.4 mg/dL (reference range: 0.2–1.3 mg/dL), albumin 2.8 g/dL (reference range: 3.5–4.7 g/dL), and INR 1.06 (reference range: 0.9–1.2) (Fig. 3; Fig. 4).

However, just 4 days after discharge from the hospital, there was recurrence of worsening diarrhea associated with nausea and emesis despite being on prednisone 40 mg/d, which raised the concern for steroid-refractory ICPI-associated enterocolitis. Anti-tumor necrosis factor (anti-TNF) biologic therapy, infliximab (5 mg/kg), was administered once. After completion of a total of 8 days of prednisone 40 mg/d, a steroid taper regimen was implemented from prednisone 30 mg/d for 3 days, 20 mg/d for 3 days, and 10 mg/d for 3 days (Fig. 1).

Outpatient labs obtained 6 days after the infliximab administration revealed interval change in liver profile, with increase in serum ALT to 62 U/L, AST to 51 U/L, and total bilirubin to 0.9 mg/dL (Fig. 3; Fig. 4).

At 29 days after the initial infliximab administration, the patient presented to the emergency room with new-onset jaundice without abdominal pain, nausea, emesis, or fever. Liver enzymes from 2 days prior to this presentation showed abrupt elevations, with serum ALT 364 U/L, AST 279 U/L, ALP 680 U/L, and total bilirubin 6.4 mg/dL. Repeat lab testing in the emergency room showed ALT 291 U/L, AST 214 U/L, ALP 677 U/L, total bilirubin 7.5 mg/dL, direct bilirubin 6.6 mg/dL, and albumin 3.5 g/dL (Fig. 3; Fig. 4). The patient did not report significant acetaminophen use or introduction of new medications.

On physical examination, he had a temperature of 37.4 °C, pulse of 80 beats per minute, blood pressure of 119/65 mmHg, respiratory rate of 16/min, and normal oxygenation on ambient air. His weight was 63 kg with a BMI 22.6 kg/m^2^. He exhibited conjunctival icterus and jaundice of the skin. The abdomen was soft without tenderness or hepatosplenomegaly. There were no stigmata of chronic or advanced liver disease. He had no signs of hepatic encephalopathy.

Lab testing for liver disease were performed (Table 1). Anti-nuclear antibody titer, anti-smooth muscle antibody, anti-liver-kidney-microsomal antibody were negative. Active viral hepatitides A, B, C, and E were excluded. CMV IgG was previously positive prior to infliximab administration, with subsequent IgM testing that was equivocal; testing for EBV IgM was negative with positive nuclear antigen and IgG; and testing for HSV-1 and HSV-2 (quantitative assays) were negative (undetectable viral load). *HFE* gene testing revealed one mutation of H63D, which was not deemed clinically significant. Additional lab results are reported in Table 1.

**Table 1:** Laboratory data

Variable	Reference Range, Adults^a^	Result
White blood cell count (per μL)	4000–11,000	5200
Hemoglobin (g/dL)	14.0–18.0	13.0
Mean corpuscular volume (fL)	82–98	87
Platelet count (per μL)	140,000–440,000	208,000
Sodium (mEq/L)	136–145	143
Blood urea nitrogen (mg/dL)	8–20	16
Creatinine (mg/dL)	0.70–1.30	1.19
Lactate dehydrogenase (U/L)	313–618	870
Hepatitis A IgM antibody	Negative	Negative
Hepatitis B surface antigen	Non-reactive	Non-reactive
Hepatitis B surface antibody (mIU/mL)	< 5.0 (Negative)	< 5.0 (Negative)
Hepatitis B total core antibody	Non-reactive	Non-reactive
Hepatitis B DNA (IU/mL)	Undetected	Undetected
Hepatitis C antibody	Non-reactive	Non-reactive
Hepatitis E IgM antibody	Negative	Negative
Hepatitis E IgG antibody	Negative	Positive
CMV IgM (units)	0.0–0.9	0.9 (Equivocal)
CMV IgG (units)	0.00–0.79	11.90
EBV viral capsid antigen IgM antibody	Negative	Negative
EBV viral capsid antigen IgG antibody	Negative	Positive
EBV nuclear antigen IgG antibody	Negative	Positive
HSV-1 DNA (copies/mL)	Not detected	Not detected
HSV-2 DNA (copies/mL)	Not detected	Not detected
Anti-nuclear antibody titer	< 1:40	< 1:40
Anti-smooth muscle antibody	Negative	Negative
Anti-mitochondrial antibody	Negative	Negative
Anti-liver kidney microsomal type 1 antibody	Negative	Negative
Ceruloplasmin (mg/dL)	19.0–31.0	30.5
Ferritin (ng/mL)	30–400	638
Iron (μg/dL)	49–181	51
Total iron binding capacity (μg/dL)	250.0–450.0	247.7
*HFE* gene mutation analysis	–	One copy of H63D detected
Triglycerides (mg/dL)	< 150	166^b^
LDL-cholesterol (mg/dL)	< 100	74^b^
Anti-tissue transglutaminase IgA antibody (U/mL)	< 4.0	< 1.2
Anti-tissue transglutaminase IgG antibody (U/mL)	< 6.0	< 1.2

^a^Reference values are affected by variables including patient population and laboratory methods employed

^b^These results were obtained over 1 year prior to presentation

### Evaluation of liver toxicity

A second CT of the abdomen and pelvis with oral and intravenous contrast demonstrated normal ileum and no major significant changes except slight increase in a sub-centimeter hypodensity in the right liver. Magnetic resonance imaging of the abdomen with magnetic resonance cholangiopancreatography showed no intrahepatic or extrahepatic biliary duct dilatation, and stable sub-centimeter densities in the liver.

**Fig. 5:**
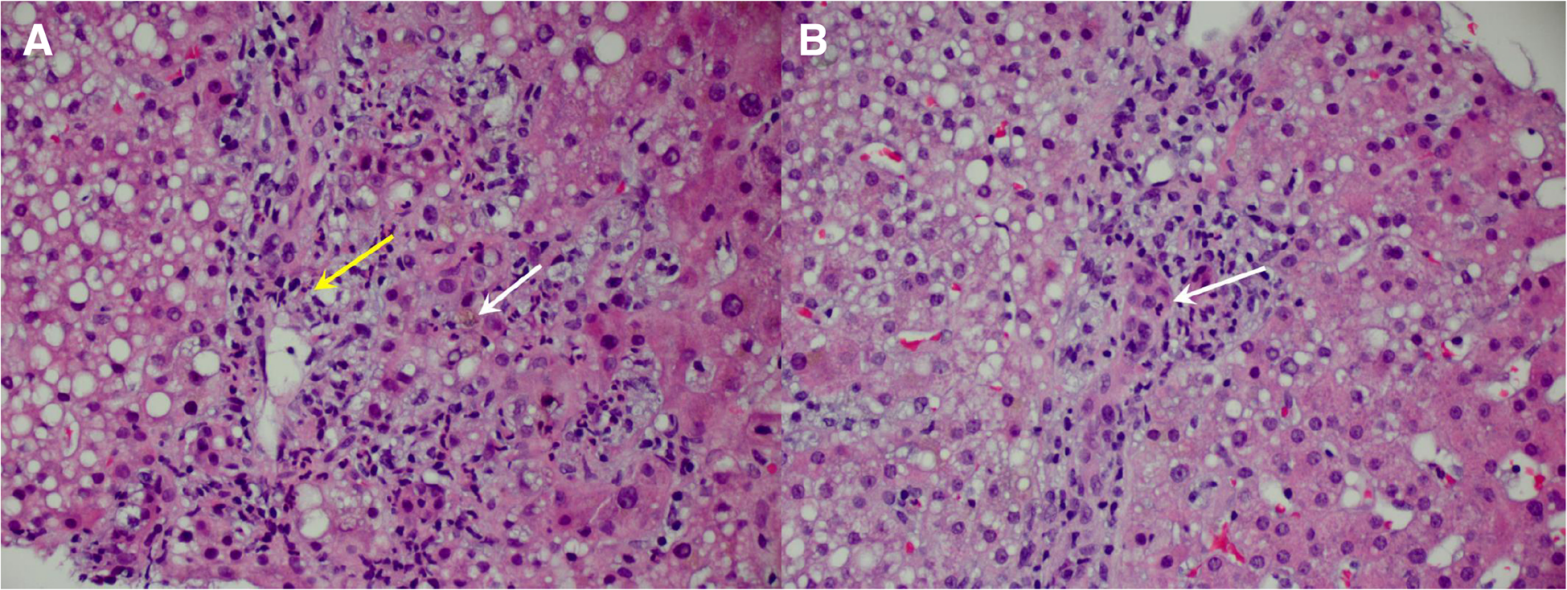
Liver biopsy histology. Panel A: (Hematoxylin and eosin stain, 20×) Portal mixed inflammation (yellow arrow) and periportal cholestasis (white arrow). Panel B: (Hematoxylin and eosin stain, 20×) Bile ductular injury or cholangitis

Due to persistent elevation of liver enzymes at 5 weeks from infliximab administration, a non-targeted liver biopsy was performed that demonstrated cholestatic hepatitis with bile duct injury, cholestasis, mixed steatosis but predominantly microvesicular steatosis (Fig. 5). There were findings of mixed inflammatory infiltrates, mild portal and periportal fibrosis, and no iron or α-1 antitrypsin cytoplasmic globules.

Intravenous methylprednisolone 1 mg/kg every 12 h were initiated for 48 h before discontinuation upon recognition of histology findings and lack of improvement of liver enzymes. By exclusion of other etiologies, and occurrence of the event despite having even previously been on corticosteroid treatment, acute drug-induced liver injury secondary to infliximab was deemed to be the most likely cause of his abnormal liver biochemical results.

### Outcome of liver injury management

By the fourteenth week post-infliximab administration, serum ALT had gradually improved to 51 U/L, AST to 53 U/L, ALP to 506 U/L, and total bilirubin to 2.2 mg/dL (Fig. 3; Fig. 4) with plans for periodic monitoring of liver enzymes. The patient’s diarrhea symptoms completely resolved after the first infusion of infliximab.

## Discussion

As ICPI therapies are being more frequently employed to treat many advanced cancers, hepatotoxicity is being recognized as one of the irAEs [1–13]. New elevation in liver transaminases during the ICPI treatment should prompt investigation of potential drug-induced liver injury. However, other co-existing conditions and/or infections that require additional medical treatments could also be associated with hepatotoxicity, the differential diagnosis toward the etiology may be confounding. This diagnostic challenge may be overcome by careful review and analysis of the history. Points of consideration include evaluation for acute viral hepatitides, autoimmune disease, alcohol-associated hepatitis, immune-related adverse event on the liver (especially given prior irAE associated with skin rash and colitis), and potentially infliximab-associated hepatitis.

Ipilimumab and nivolumab have been independently associated with the liver irAE [3–13]. The incidence of hepatoxicity for ipilimumab alone, for instance, was estimated to be 1.6–3.9% (ascertained by ALT elevations) in any grade of severity [7, 11]. Grade 3 or 4 hepatotoxicity has an estimated 3–7% incidence [3]. Recent data suggests an incidence of hepatotoxicity of as high as 11%, with a reported fatality rate of 0.2% [13]. Liver injury as a result of exposure to ipilimumab usually occur after 2–4 cycles or a median time of 3–9 weeks after initiation of the drug, with potential to cause elevations in liver tests to reflect hepatocellular or mixed cholestatic injury [3–5]. Nivolumab may also cause hepatotoxicity, with onset of injury 2–6 cycles or 1–3 month after initiation of treatment [6]. The incidence of ALT elevations from nivolumab alone is estimated to be up to 3.8% in any grade of severity, based on clinical trial data [7]. The pattern of liver injury from nivolumab resembles that from ipilimumab. Varied findings may result on abdominal imaging of the liver (CT or ultrasound) from normal findings to mild hepatomegaly, periportal edema, and low-attenuation of the liver parenchyma [14]. Combination therapy utilizing both a CTLA-4 inhibitor and PD-1 inhibitor have been applied towards advanced melanoma treatment and is also being explored in other cancers at the expense of higher risk for irAE [15, 16]. Recent data estimates hepatotoxicity from combined therapy to be about 13% with median time to onset of about 2.1 months [13]. Prior clinical trial data had suggested the overall incidence of ALT elevations in combined therapy to be as high as 17.6% [7]. Hepatocellular and/or cholestatic liver injury patterns may be seen [17]. There is no compelling relationship between the autoimmune antibodies and autoimmune liver disease [5, 17, 18].

Histopathological findings of ICPI-related hepatitis had been reported previously. Such features included: lymphohistiocytic inflammation with interface hepatitis, eosinophilic infiltration or acidophil bodies, granulomas, perisinusoidal fibrosis, periportal fibrosis, and/or biliary ductal injury [9, 14, 17, 33]. While there is no known pathognomonic histologic finding to distinguish ICPI-related hepatitis from histologic findings generally seen in drug-induced liver injury (DILI), granulomas, with or without fibrin deposits, and variable presence of plasma cells, continue to be characterized in more recent cases of ICPI-related hepatitis [17].

The current standard initial treatment for ICPI-related hepatitis is immunosuppressive therapy with high-dose corticosteroids such as methylprednisolone or prednisone [7, 8, 11, 16, 17]. In a recent study, few patients were documented to have achieved spontaneous remission without corticosteroid therapy [17].

Infliximab is an anti-TNF-α inhibitor that has been well-studied in treating inflammatory bowel disease. Published reports of its application in this context were reported since 2006 [19], and subsequent applications of its use to treat ipilimumab-induced colitis followed [20–24], where infliximab has demonstrated efficacy in providing prompt remission in diarrhea symptoms. Infliximab-associated liver injury remains a potential concern in such patients [25–30]. The incidence of infliximab-associated liver injury was estimated to be 0.68-0.83% in prior studies examining DILI [29, 30]. This adverse effect may present in several potential forms: hepatocellular injury; cholestatic liver injury, autoimmune-like, or reactivation of viral hepatitis B [26, 28, 31, 32]. Unless jaundice manifests, cases are generally asymptomatic. Liver enzymes are generally monitored periodically while on infliximab therapy.

Despite alcohol use and prior administration of three cycles of ipilimumab and nivolumab, this particular patient’s liver biochemical profile was unremarkable and stable 4 months after initiation of the immune agents. The first sign of ALT elevation occurred only 6 days after the first infusion of infliximab and had increased six-fold by 4 weeks. Hepatitis occurred despite being on prior steroid therapy prescribed for his treatment of colitis. A brief period of high-dose intravenous steroids for presumed ICPI-related hepatitis did not improve the liver enzymes elevation. Overall, ALT levels remained significantly elevated above 200 U/L until 8 weeks post-infusion of infliximab when ALT levels began to decline. There was a mixed cholestatic pattern of liver injury with peak alkaline phosphatase levels as high as 1852 U/L by 8 weeks post-infusion, and direct hyperbilirubinemia with total bilirubin as high as 20.7 mg/dL by 7 weeks post-infusion. This patient did not have hepatitis B infection nor develop any pertinent autoimmune markers. Although EBV and CMV DNA PCR were not specifically tested, the patient’s clinical condition did not eventually worsen in the absence of antiviral therapy, despite the new addition of immunosuppressive therapy.

Cases of ICPI-associated hepatitis, AIH, and other forms of DILI were compared in a previous histologic study that suggested expectation of less confluent necrosis compared to both AIH and DILI, less plasmacytosis compared to AIH, and less eosinophilic infiltration than in DILI, but no difference in portal tract inflammation [9]. The liver biopsy of this patient revealed inflammatory infiltrates attributed to acute hepatitis, whereas findings of steatosis and mild fibrosis may be related to subtle chronic liver disease in the setting of chronic alcohol use. Since the current liver histologic knowledge was insufficient to differentiate ICPI-associated liver injury from other forms of DILI, additional understanding of the pathophysiology of irAE in conjunction with the analysis of the clinical timeline led to a clinical judgment towards attribution of the patient’s liver injury to infliximab exposure.

## Conclusion

Diarrhea is a well-known side effect of ICPIs that often requires the use of steroids to induce remission. In steroid-refractory cases, biologic agents such as infliximab have been increasingly employed with good therapeutic effect. Since future patients are likely to encounter such a clinical scenario, hepatotoxicity from infliximab must be carefully monitored. The differential of acute liver injury in this patient population will become more expansive. Therefore, careful evaluation of the pharmacologic chronology and prompt exclusion of other potential acute liver disease, including acute viral hepatitis and immune-related liver injury, are important. While liver histology itself may not necessarily distinguish the causative agent, it may help exclude other known forms of liver disease and assess the degree of liver injury.
